# Persistence and accumulation of environmental DNA from an endangered dragonfly

**DOI:** 10.1038/s41598-021-98099-1

**Published:** 2021-09-23

**Authors:** Kristie J. Schmidt, Daniel A. Soluk, Sarah E. Mays Maestas, Hugh B. Britten

**Affiliations:** University of South Dakota, Vermillion, SD USA

**Keywords:** Conservation biology, Freshwater ecology, Molecular ecology

## Abstract

Detection of environmental DNA (eDNA) has become a commonly used surveillance method for threatened or invasive vertebrates in both aquatic and terrestrial environments. However, most studies in this field favor vertebrate target species. Environmental DNA protocols can be especially useful for endangered invertebrates such as the Hine’s emerald dragonfly (*Somatochlora hineana*) where conservation efforts have been greatly hindered by training, time, overall costs, and environmental impacts associated with conducting surveys in the calcareous fens occupied by this species. An essential step in developing such a protocol is to evaluate the dynamics of eDNA concentration under controlled conditions. We used the quantitative polymerase chain reaction (qPCR) to examine seasonal shifts in the persistence and net-accumulation of eDNA from captive *S. hineana* larvae in experimental mesocosms at temperatures corresponding with their overwintering (5.0 °C) and active (16.0 °C) seasons. Environmental DNA persisted longer at 5.0 °C but accumulated more readily at 16.0 °C. Differences in the accumulation and persistence of eDNA reflect differences in the longevity of eDNA at different temperatures and seasonal differences in larval *S. hineana* behavior. This study highlights the importance of considering how seasonal changes in temperature influence not only the speed of eDNA degradation but also the target species’ eDNA shedding rates.

## Introduction

We developed environmental DNA (eDNA) detection protocols to assist in habitat identification for conservation for the US federally endangered Hine’s emerald dragonfly (*Somatochlora hineana*). Larval *S. hineana* have been observed in groundwater-fed calcareous fen habitats in Illinois, Wisconsin, Michigan, and Missouri in the USA, and Ontario, Canada. Habitat destruction and fragmentation have been the primary cause of *S. hineana* population decline^1^. Therefore, a key part of conservation efforts to benefit *S. hineana* is the identification and protection of any remaining habitat areas. Conventional sampling for the presence of *S. hineana* often includes both adult and larval sampling.

Larval *S. hineana* surveys include benthic-sampling and the pumping of crayfish burrows. Larval *S. hineana* are most often found in the burrows of *Cambarus *(= *Lacunicambarus*)* diogenes* throughout the year and are almost exclusively found in *C. diogenes* burrows during their overwintering period^2^. Comprehensive larval surveys can take months to complete, require intensive training of field personnel, are reliant on favorable weather conditions, and are only effective if late instar larvae can be collected for identification. Adult *S. hineana* surveys are difficult due to short flight season, habitat segregation by sex, large potential flight range (adults can range for many kilometers from larval habitat), risk of harm when netting adult dragonflies, and difficulty observing genitalia characteristics necessary for accurate species identification when in flight^1^.

Given the restrictions of conventional sampling techniques, there has been a great need to develop a method to expedite field site identification. Environmental DNA can be used to guide and prioritize locations for conventional surveying methods, increasing the speed at which habitats can be identified for protection and restoration.

Environmental DNA (eDNA) is a relatively new surveillance method used to detect the presence of a species within a habitat by collecting environmental samples (e.g., soil and water) that contain cell fragments and exogenous DNA^3^. Mitochondrial genes, which are more plentiful and have a higher resistance to degradation than nuclear genes, are targeted and amplified to determine species presence or absence^4–7^.

Currently, there is a taxonomic skew toward fish, amphibian, and mollusk eDNA studies^7,8^ suggesting the need to determine if eDNA methods can be useful for detecting aquatic insects. Environmental DNA analysis from 27 taxa of freshwater arthropods had been published as of 2019; some of these taxa include *Procambarus clarkii*, *Pacifastacus leniusculus*, and *Gammarus pulex*^8^. Additionally, the critically endangered plecopteran *Isogenus nubecula* was detected using eDNA methods^9^.

The potential advantages of using eDNA rather than traditional surveying methods include the reduction of field labor hours^10^, reduced impact to sensitive habitats^7^, and a lower threshold of detection^11,12^. Additionally, eDNA has proven to be an effective tool when traditional methods require timely/costly surveying efforts^6^ and for detecting cryptic invasive species^10^.

Although there is always some risk of damaging the habitat when studying a system, environmental DNA sampling (i.e., water, soil, ice) is much less invasive and has far less potential for harming native and endangered species than many traditional surveying methods^7^. For example, electrofishing can cause damage in the form of removing/killing fish from the sample site^13^. Traditional sampling methods for larval populations of *S. hineana* include benthic sampling (monitoring populations in stream beds) and burrow-pumping (a novel technique used to locate larvae within crayfish burrows)^2^. These techniques can disrupt flow patterns within shallow streams, collapse burrows, and harm/kill sampled individuals.

While there has been some speculation that eDNA sampling may have high false-positive rates due to ancient DNA contamination from extirpated populations, studies show that eDNA typically becomes undetectable in water within 1–44 days after source removal^10,14–21^ and approximately 144 days in soil^22^. This suggests that eDNA surveys are contemporaneous and can be used to inform conservation efforts.

Environmental DNA degradation is likely more complex in a field setting, and the *persistence* (defined here as the length of time eDNA remains detectable within a habitat or mesocosm) and *net-accumulation* (defined here as the difference between the amount of eDNA produced and the amount of eDNA degraded over time) are likely to vary depending on numerous factors that alter source/sink dynamics^3^. Spatiotemporal dynamics are especially important in affecting the persistence and accumulation of eDNA in the field and need to be accounted for when developing eDNA methodologies^23^. Concentrations of eDNA may fluctuate spatially and/or temporally as a result of fluctuations in biomass^18,24,25^, transport through a flowing system^17,26–28^, age structuring of target populations^7,16^, feeding activity^29^, life-history events^5^, seasonal habitat preference^13,30^, water temperature^24,31–33^, hydrology^13,27^, inhibition^13,27^, and microbial activity^34^. Some studies show that water pH affects eDNA degradation rates^19^, while others do not^35^. Similarly, some studies show that UV light exposure affects eDNA degradation rates^17^, while others show no such effect^36^.

In this study, we focused on the effects that seasonal shifts in temperature have on the persistence and net-accumulation of larval *S. hineana* eDNA. Since temperature drives the production of eDNA through metabolic processes^31^ and directly alters the rate of microbial degradation of eDNA^32^, it may be the most important variable driving seasonal shifts in eDNA detection.

*Somatochlora hineana* larval molting activity varies with seasonal changes, the net-accumulation of *S. hineana* eDNA within a habitat. Adult *S. hineana* females lay eggs within streams and streamlets during their flight period (July–early August). Eggs typically mature over winter. In the following year, hatching of pro-larva from eggs occurs between April and June. All *S. hineana* larvae go through approximately 12 larval instars (F-11 to F-0). The first 6 larval instars (F-11 through F-6) occur rapidly within the first year, and the final 6 (F-5 through F-0) occur more slowly over a period of 2–4 years^1^. Since *S. hineana* larvae take several years to fully mature, they survive overwintering in shallow, partially frozen streams within *Cambarus *(= *Lacunicambarus*)* diogenes* crayfish burrows. While *S. hineana larvae* overwinter within burrows, they rarely consume food or molt, thus reducing the amount of eDNA shed^2^.

The net-accumulation of larval *S. hineana* eDNA was likely to increase with increasing temperatures^2,31,37^, while the persistence of larval *S. hineana* eDNA was likely to decrease with increasing temperatures^32^. Therefore, we assessed the seasonal shift in persistence and net-accumulation of larval *S. hineana* eDNA in temperature-controlled mesocosms that reflect the larval overwintering period (5.0 °C) and the larval active period (16.0 °C). This study provided preliminary information regarding the seasonal shift in eDNA production for larval *S. hineana*. Understanding the seasonal dynamics of larval *S. hineana* eDNA is vital for efficient detection of this rare aquatic species using eDNA protocols. Our mesocosm results have informed subsequent field sampling of *S. hineana e*DNA.

## Results

### Quality control

No extraction blank or no-template-control tested positive, indicating no contamination. All positive and spiked positive controls tested positive, indicating no inhibition or reagent failure. Average standard curve r^2^ values, which measure replicate reproductivity, were r^2^ = 0.96 and r^2^ = 0.98 for CYTb/FAM and COX3/HEX, respectively. Average amplification efficiency, determined by the slope of the standard curve, was 97.76% and 98.61% for CYTb/FAM and COX3/HEX, respectively. The limit of detection (LOD) for CYTb and COX3 with four sample replicates was 2 copies/$\upmu$L and 20 copies/$\upmu$L respectively (unpublished data).

### Net accumulation of *S. hineana* eDNA

At 16.0 °C, containers holding *S. hineana* larvae in the large size class (approx. 0.33 g) accumulated significantly more eDNA than those with larvae in the medium size class (approx. 0.06 g) (linear mixed effects, n = 64, df = 375, p < 0.01) and in the small size class (approx. 0.01 g) (linear mixed effects, n = 64, df = 375, p < 0.001). Containers holding small and medium size class larvae accumulated comparable amounts of eDNA (linear mixed effects, n = 64, df = 375, p = 0.20). At 5.0 °C, containers holding larvae in the medium size class accumulated significantly more eDNA than those containers holding larvae in the large size class (linear mixed effects, n = 64, df = 375, p = 0.04). There was no difference in the accumulation among large and small (linear mixed effects, n = 64, df = 375, p = 0.45) or small and medium (linear mixed effects, n = 64, df = 375, p = 0.19) larval size classes.

**Figure 1:**
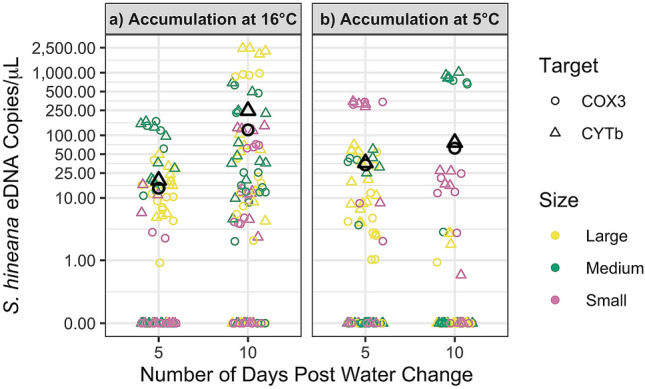
Significant accumulation (p < 0.001) in *S. hineana* eDNA concentrations at 16 °C between 5 and 10 days post water change. No significant change in *S. hineana* eDNA concentrations at 5 °C between 5 and 10 days post water change (p = 0.37). Shape indicates gene target (COX3, CYTb). Black shapes indicate average quantification for each target. Red, green, and blue shapes indicate quantifications for large, medium, and small size classes, respectively. Points are graphed on a pseudo-log scale (0.1–2500) with break points at (0, 1, 10, 25, 50, 100, 250, 500, 1000, and 2500).

At 16.0 °C, the concentration of *S. hineana* eDNA increased significantly between days 5 and 10 (linear mixed effects, n = 96, df = 375, p < 0.001; Fig. 1a; Table 1). At 5.0 °C, there was no significant change in concentration of *S. hineana* eDNA between days 5 and 10 (linear mixed effects, n = 64, df = 375, p = 0.37 Fig. 1b; Table 1).

**Table 1:** Average *Somatochlora hineana* eDNA concentrations—accumulation experiment.

Treatment	Target	Day 5	Day 10
Net accumulation 16.0 °C	COX3:	14.31 copies/μL	122.04 copies/μL
	CYTb:	19.07 copies/μL	247.76 copies/μL
Net accumulation 5.0 °C	COX3:	33.65 copies/μL	62.10 copies/μL
	CYTb:	36.25 copies/μL	76.50 copies/μL

### Persistence of *S. hineana* eDNA

**Figure 2:**
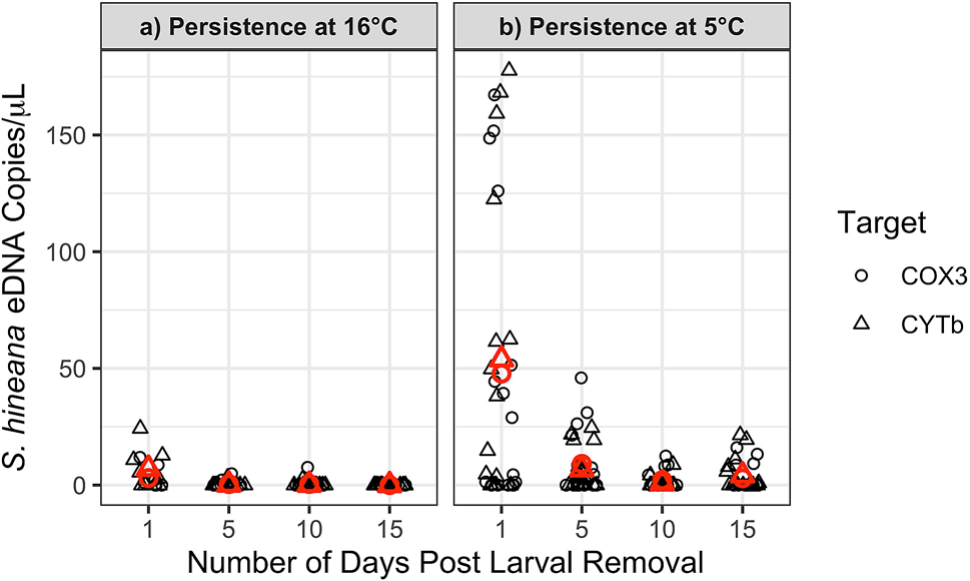
Significant decrease in *S. hineana* eDNA concentrations at 16 °C (p < 0.01) and 5 °C (p < 0.001) between 1 and 15 days post larval removal. Shape indicates gene target (COX3, CYTb). Red shapes indicate average quantification for each target.

**Figure 3:**
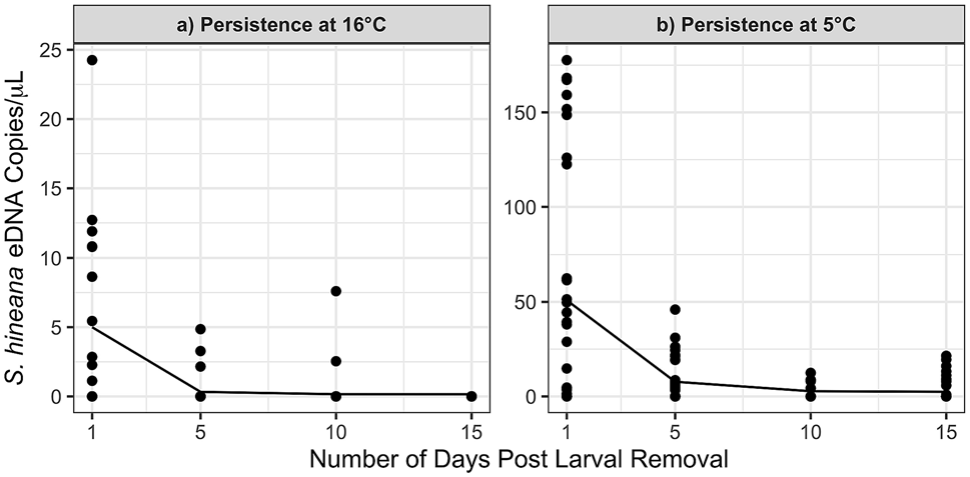
Exponential decay of *S. hineana* eDNA at 16 °C ($\alpha =0.83)$ and 5 °C ($\alpha =0.55)$ days 1, 5, 10, and 15 post larval removal. Note: parts (**a**) and (**b**) display different y-axes.

At 16.0 °C, average concentrations of *S. hineana* eDNA significantly decreased between days 1 and 15 (linear mixed effects [ln(Quantity + 1)], day 1 n = 16, day, day 15 n = 32, df = 232, p < 0.01; Fig. 2a; Table 2). Larval *S. hineana* eDNA decayed exponentially with an alpha decay rate of 0.83 (Fig. 3a). At 5.0 °C, the average concentration of *S. hineana* eDNA significantly decreased between days 1 and 15 (linear mixed effects [ln(Quantity + 1)], n = 32, df = 232, p < 0.001; Fig. 2b; Table 2). Larval *S. hineana* eDNA decayed exponentially with an alpha decay rate of 0.55 (non-linear least squares model, Fig. 3b).

**Table 2:** Average *Somatochlora hineana* eDNA concentrations—persistence experiment.

Treatment	Target	Day 1	Day 5	Day 10	Day 15
Persistence 16.0 °C	COX3:	2.99 copies/μL	0.44 copies/μL	0.47 copies/μL	0.00 copies/μL
	CYTb:	7.01 copies/μL	0.20 copies/μL	0.16 copies/μL	0.00 copies/μL
Persistence 5.0 °C	COX3:	47.79 copies/μL	9.07 copies/μL	2.09 copies/μL	2.94 copies/μL
	CYTb:	53.90 copies/μL	6.75 copies/μL	0.81 copies/μL	4.16 copies/μL

## Discussion

This study of *S. hineana* eDNA persistence and net-accumulation provides an example of one of the first applications of using emerging eDNA detection technology for the conservation of an endangered dragonfly (Odonata). We studied seasonal shifts in eDNA concentrations under controlled conditions at environmentally relevant temperatures. Our eDNA mesocosm results have been used to plan the timing of field sampling in our *S. hineana* study areas in Illinois and Wisconsin, two areas with different annual temperature profiles.

Although organisms like fish readily shed mucous and/or epithelial cells during periods of inactivity, organisms with an exoskeleton are more conservative in eDNA shedding rates, making them more difficult to detect^12^. The primary source of eDNA from living *S. hineana* is from molting and the production of fecal matter^37^. Our results indicate that overwintering larvae rarely shed eDNA. Additionally, eDNA persists longer at overwintering temperatures but has greater net-accumulation at temperatures when larvae are active. Therefore, eDNA sampling for larval *S. hineana* populations is likely more efficient in the spring/summer than in the fall/winter.

Results from our persistence experiment are consistent with other studies indicating that eDNA does not persist in a system beyond several weeks^10,14,16,20^, and that eDNA degrades more quickly at higher temperatures^24^. These results are also consistent with other eDNA studies indicating that eDNA degrades exponentially over time^4,17,18^. Our results indicated that eDNA signals are contemporaneous, reflecting recent eDNA production.

When water temperatures are near 5.0 °C, *S. hineana* eDNA signals can be detected 2 weeks after shedding. However, our exponential decay curve (Fig. 3b) suggested that eDNA concentrations neared the limit of detection for CYTb within 10 days (LOD: 2 copies/$\upmu$L) and within 5 days for COX3 (LOD: 20 copies/$\upmu$L). This suggests that larval *S. hineana* eDNA is unlikely to persist at a detectable level in the environment after 2 weeks, even at overwintering temperatures. Therefore, assuming this pattern applied to field-collected eDNA samples, larval *S. hineana* eDNA results are contemporaneous.

Previous studies indicated that eDNA shedding rates can vary among older/younger and smaller/larger individuals. Some studies suggest that smaller individuals produce more eDNA than larger individuals due to rapid rates of development and molting^12^, while other studies suggest that eDNA production increases with biomass^18,24^. Our results showed that variation in eDNA shedding rates were season- or temperature dependent. At 16.0 °C, large larvae produced more eDNA than medium or small larvae, suggesting that eDNA production increases with biomass. However, at 5.0 °C medium larvae produced more eDNA than large larvae, indicating that medium sized individuals were more metabolically active than larger individuals while overwintering.

Consistent with studies indicating that eDNA shedding rates increase with higher temperatures^24,33^, *S. hineana* eDNA accumulated more readily over time in our 16.0 °C mesocosms than in our 5.0 °C mesocosms. Just as behavioral changes in other invertebrate species affect eDNA shedding rates^16^, the decreased rate of eDNA production by *S. hineana* larvae is likely associated with the reduction of activity among *S. hineana* larvae later in the season at overwintering temperatures. Preliminary field sample analysis suggests that our qualitative predictions of seasonal eDNA concentrations deduced from these mesocosm experiments are borne out in *S. hineana* habitat^38^.

These results suggest that it is more important to consider the effects that seasonal changes, including temperature, have on behavior (e.g., molting and overwintering) rather than the effects of temperature on eDNA persistence (e.g., microbial decay) when planning efficient eDNA sampling periods. Determining the most efficient time to detect target DNA is especially important for organisms with an exoskeleton, which generally produce small quantities of eDNA^7,8,12^. Given this, we expect that field detection of larval *S. hineana* eDNA will be less efficient when water temperatures are low enough to induce overwintering behaviors.

However, additional life history characteristics such as emergence have also been shown to impact seasonal eDNA concentrations^5^. Therefore, we predict that summer (after hatching and prior to emergence) would be the most efficient time to detect *S. hineana* eDNA in the field*.* This trend likely describes eDNA detection efficiency for all aquatic invertebrates with life-histories similar to *S. hineana*.

To test this, we developed field experiments to document temporal variation in eDNA concentration over several months at sites with known *S. hineana* occupancy and detected eDNA concentrations comparable to those reported in this study^38^. For field samples, we increased sample volumes to reduce the risk of false-negative results when sampling from a highly dilute environment^36^. Since one study showed that qPCR methods were ineffective at detecting eDNA from endangered aquatic insects due to inhibition^9^, we continued to monitor for inhibition in field samples using “spiked” controls containing a mixture of sample and positive control template^38^.

During efficient sampling periods (e.g., summer months), eDNA can be a useful tool in the identification of *S. hineana* habitat. Environmental DNA results can be used to prioritize sites for traditional surveying methods, significantly increasing the efficiency of habitat detection and assessment. Similar eDNA projects may also be beneficial in habitat identification of many other threatened, endangered or invasive aquatic invertebrates.

## Methods

Larvae used for this study had been reared from eggs over the course of 1–3 years from *S. hineana* females obtained at sites in Wisconsin and Illinois as part of a captive rearing program. All larvae and eggs of *S. hineana* were handled according to Federal Fish and Wildlife Native Endangered Species Permit (TE805269-15) issued to D. Soluk. Once larvae reached their 6th instar (F-5, F-6), they were individually held in 120 mL capacity specimen cups that were filled with approximately 80 mL of water. Larvae were kept in oxygenated water that was collected from a shallow well in Door County, WI. This water was similar to the groundwater that feeds larval habitat areas but had never come into contact with *S. hineana* DNA prior to addition to the specimen cups.

In the fall, the larvae were kept at 16.0 °C and were regularly fed a diet of amphipods and larval chironomids. Larvae were given regular water changes every 2–6 weeks. At experimental day 0 for the 16.0 °C trial, water was removed from specimen cups, combined, placed into beakers for the persistence experiment, and replaced with fresh water. Following the experiments at 16.0 °C, the water surrounding the larvae was adjusted over a period of 2–3 weeks down to 5 °C leading up to *S. hineana*’s overwintering period. Since we have observed that *S. hineana* larvae do not consume prey while overwintering at 5 °C, larvae at this temperature were not fed. At experimental day 0 for the 5.0 °C trial, water was removed from specimen cups, combined, placed into beakers for the persistence experiment, and replaced with fresh water. Experiments were conducted in this manner two years in a row. Sixty milliliter water samples were taken with a syringe and immediately filtered through 0.45 $\upmu$m 25 mm Whatman Nitro-Cellulose Filters for future eDNA extraction. Specimen cups and beakers were gently nutated prior to sample collection to homogenize eDNA concentration throughout the container.

### Net accumulation sample collection

To measure the net-accumulation of *S. hineana* eDNA at 16.0 °C and 5.0 °C, we filtered 60 mL water samples from larval specimen cups 5 and 10 days post water change. Six larvae were selected on each collection day to provide samples. Two larvae from each of three size classes were chosen: large (instar class F-0 to F-1, approximately 0.33 g live mass), medium (instar class F-2 to F-3, approximately 0.06 g live mass), and small (instar class F-4 to F-6, approximately 0.01 g live mass). Different larvae were chosen at days 5 and 10 post water change. Each larval specimen cup was only sampled once during this experiment.

### Persistence sample collection

To measure persistence of *S. hineana* eDNA at 16.0 °C and 5.0 °C, water and debris removed from all specimen cups during the water change at day 0 of the accumulation experiment were combined and placed in one of two beakers. The two beakers were considered replicates and stored in identical conditions. At days 1, 5, 10, and 15 post larval removal, 60 mL water samples were taken from each beaker which contained approximately 600 mL of water. Filtered water was not returned to the beaker after sampling.

### Sample extraction

We extracted sample filters using the QIAGEN DNeasy Blood and Tissue Kit commonly used for eDNA studies^17,25^. Approximately ½ of each filter was extracted, and the other ½ of each filter was stored in 75% ethanol at room temperature as a voucher. For every set of extractions, one extraction blank using only reagents and no sample was also processed to ensure that the reagents used were not contaminated. The ½ of the filter intended for extraction was further cut into thirds to increase lysis buffer exposure throughout the filter. These pieces were placed in open microcentrifuge tubes and allowed to dry in a fumigation hood until all of the ethanol had evaporated from the sample (approximately 6–12 h). Two alterations were made to the QIAGEN Spin Column—Animal Tissue Protocol: (1) QIAGEN Mini-Prep Spin Columns were used after sample lysis and before the precipitation of DNA with Buffer AL and 100% ethanol; (2) Samples were eluted using 100 μL of elution buffer twice for an end volume of 200 μL.

### qPCR

Environmental DNA primers, probes, and g-block specific to *S. hineana* were developed at the US Geological Survey’s Upper Midwest Environmental Science Center based on the dragonfly’s full mitochondrial genome^39^. Primer/probe sets specific to *S. hineana* mitochondrial DNA sequences Cytochrome c oxidase subunit 3 (COX3: 139 base pairs [bp]) and Cytochrome b (CYTb: 125 bp) (Table 3) were multiplexed using qPCR^39^. HEX-labeled probes were used in conjunction with COX3 primers, and FAM-labeled probes were used in conjunction with CYTb primers. The limit of detection for these primers was approximately 20 copies/$\upmu$L (unpublished data).

**Table 3:** Primer/probe sequences used to detect *Somatochlora hineana* eDNA.

Primer/probe	Forward primer	Reverse primer	Probe
Cytochrome c oxidase subunit 3 (COX3)/HEX	GCTCCATTCACTATTGCAGATTC	GTGGTGAGAAGTGGCTTATGT	AGCAACTGGATTTCATGGAATTCACGT
Cytochrome b (CYTb)/FAM	GCAGCTGCTACAATAATTCAC	CCATGAGAAATATGGATGGAAAG	CATCAAACTGGTTCCAATAACCCAATTGGT

The primers and probes used to detect *S. hineana* for this study were cross-validated against several species of *Somatochlora* (*S. minor*, *S. elongata*, *S. franklini*, *S. incurvata, S. kennedyi, S. walshii, S. williamsoni, S. tenebrosa, S. linearis,* and *S. ensigera*) as well as several aeshnid and libellulid species (unpublished data). Furthermore, water samples from three locations outside the known range of *S. hineana,* which support numerous dragonfly species that are sympatric with *S. hineana,* were tested with qPCR for the presence of *S. hineana* primer, probe, and g-block eDNA sequences. All these samples were negative for the presence of the *S. hineana* mtDNA sequences used in this study (unpublished data).

Each sample was run in quadruplicate with both primer/probe sequences for a total of eight possible amplifications per sample. Each sample replicate consisted of a 20 μL reaction containing 10 μL of IDT’s 2 × Master Mix with hot-start DNA polymerase, a 0.5 μm concentration of each of the four primers, and 0.25 μm concentration of each probe. During the qPCR run protocol, samples were initially held at 95 °C for three minutes and then cycled 45 times through denaturation (95 °C for 15 s), annealing (1.6 °C decrease per second), and extension (55 °C for 60 s).

G-block DNA, a single stranded DNA molecule (526 bp) containing COX-3 and CYT-b sequences specific to *S. hineana* and ND 5 and CR2 sequences specific to *C. diogenes*^39^, was purchased through IDT’s custom DNA Oligos tool. The g-block was used to create standard curves for eDNA quantification by providing equimolar concentrations of all targets, allowing for unbiased amplification. One microliter of g-block DNA was run in duplicate for each plate at dilutions of 10^1^ to 10^6^ copies/$\upmu$L in 10^1^ $\upmu$L increments to produce a standard curve on each plate. Sample quantities were determined by comparison to this standard curve. The critical threshold value (Ct-value) was set at 0.1 cq for both primer/probe sets. This value determines the level of fluorescence at which the sample is quantified.

### Quality control

To prevent contamination, samples were handled using aseptic techniques. Separate rooms were used for sample preparation, extraction, and amplification. Before use, all surfaces were cleaned with a 25% bleach solution and pipettes were cleaned using DNA Away. All qPCR plates were prepared in a laminar flow hood, which was sterilized using a 20 min UV exposure immediately before and after plate preparation. To prevent sample degradation, filters were stored at room temperature out of direct sunlight in 75% laboratory-grade ethanol prior to extraction and DNA extracts were stored at – 20 °C.

To identify the presence of compounds that would inhibit the amplification of sample DNA, we ran three “spiked” positive controls for each sample. Spiked positive controls consisted of 1 μL of 10^3^ copies/μL g-block DNA mixed with 1 μL of sample template. If spiked samples had not amplified, this would have indicated the presence of inhibiting compounds. Four positive controls were run on each plate using 1 μL of previously extracted *S. hineana* DNA as sample template. One extraction blank was run for each extraction to monitor possible extraction reagent contamination. Ten no-template controls were run on each plate to monitor possible qPCR reagent contamination. Limit of Detection was calculated following standard eDNA qPCR assays^41^.

### Sample analysis

COX3 and CYTb sequence amplifications were multiplexed to increase likelihood of detection. Figures 1 and 2 were produced using the package ggplot2^40^. Figure 3 was produced using qplot from the R base package^42^. To analyze eDNA net-accumulation, we calculated the difference of least squared-means using linear mixed effects models (R^42^ lme4 package^43^) comparing eDNA quantities among both sampling day and larval size class including interactions between COX3/Cytb amplification as a source of random error. To analyze eDNA persistence, we calculated the difference of least squared-means using linear mixed effects models (R^42^ lme4 package^43^) comparing the natural log of eDNA sample quantity (+ 1 to include samples quantified at 0.00 copies/μL) among sampling dates including interactions between COX3/Cytb amplification as a source of random error. Exponential decay rate was analyzed with a non-linear least squares model (NLS)^4^. Specifically, we implemented an NLS model in R^42^ using the *SSasymp* function^44^.

## Data Availability

The datasets generated during the study are available from the corresponding author on reasonable request.

## References

[1] Foster SE, Soluk DA (2006). Protecting more than the wetland: The importance of biased sex ratios and habitat segregation for conservation of the Hine’s emerald dragonfly, *Somatochlora hineana* Williamson. Biol. Conserv..

[2] Pintor LM, Soluk DA (2006). Evaluating the non-consumptive, positive effects of a predator in the persistence of an endangered species. Biol. Conserv..

[3] Stewart KA (2019). Understanding the effects of biotic and abiotic factors on sources of aquatic environmental DNA. Biodivers. Conserv..

[4] Barnes MA (2014). Environmental conditions influence eDNA persistence in aquatic systems. Environ. Sci. Technol..

[5] Bista I (2017). Annual time-series analysis of aqueous eDNA reveals ecologically relevant dynamics of lake ecosystem biodiversity. Nat. Commun..

[6] Ficetola GF, Miaud C, Pompanon F, Taberlet P (2008). Species detection using environmental DNA from water samples. Biol. Lett..

[7] Thomsen PF, Willerslev E (2015). Environmental DNA—An emerging tool in conservation for monitoring past and present biodiversity. Biol. Conserv..

[8] Belle CC, Stoeckle BC, Geist J (2019). Taxonomic and geographical representation of freshwater environmental DNA research in aquatic conservation. Aquat. Conserv. Mar. Freshw. Ecosyst..

[9] Mauvisseau (2019). Combining ddPCR and environmental DNA to improve detection capabilities of a critically endangered freshwater invertebrate. Sci. Rep..

[10] Piaggio AJ (2014). Detecting an elusive invasive species: A diagnostic PCR to detect Burmese python in Florida waters and an assessment of persistence of environmental DNA. Mol. Ecol. Resour..

[11] Jerde CL, Mahon AR, Chadderton WL, Lodge DM (2011). “Sight-unseen” detection of rare aquatic species using environmental DNA. Conserv. Lett..

[12] Treguier A (2014). Environmental DNA surveillance for invertebrate species: Advantages and technical limitations to detect invasive crayfish *Procambarus clarkii* in freshwater ponds. J. Appl. Ecol..

[13] Erickson RA (2016). Detecting the movement and spawning activity of bigheaded carps with environmental DNA. Mol. Ecol. Resour..

[14] Dejean T (2011). Persistence of environmental DNA in freshwater ecosystems. PLoS One.

[15] Goldberg CS, Sepulveda A, Ray A, Baumgardt J, Waits LP (2013). Environmental DNA as a new method for early detection of New *Zealand mudsnails* (*Potamopyrgus antipodarum*). Freshw. Sci..

[16] Maruyama A, Nakamura K, Yamanaka H, Kondoh M, Minamoto T (2014). The release rate of environmental DNA from juvenile and adult fish. PLoS One.

[17] Pilliod DS, Goldberg CS, Arkle RS, Waits LP (2014). Factors influencing detection of eDNA from a stream-dwelling amphibian. Mol. Ecol. Resour..

[18] Sassoubre LM, Yamahara KM, Gardner LD, Block BA, Boehm AB (2016). Quantification of environmental DNA (eDNA) shedding and decay rates for three marine fish. Environ. Sci. Technol..

[19] Seymour M (2018). Acidity promotes degradation of multi-species environmental DNA in lotic mesocosms. Commun. Biol..

[20] Thomsen PF (2012). Detection of a diverse marine fish fauna using environmental DNA from seawater samples. PLoS One.

[21] Thomsen PF (2012). Monitoring endangered freshwater biodiversity using environmental DNA. Mol. Ecol..

[22] Turner CR, Uy KL, Everhart RC (2015). Fish environmental DNA is more concentrated in aquatic sediments than surface water. Biol. Conserv..

[23] Mathieu C (2020). A systematic review of sources of variability and uncertainty in eDNA data for environmental monitoring. Front. Ecol. Evol..

[24] Jo T, Murakami H, Yamamoto S, Masuda R, Minamoto T (2019). Effect of water temperature and fish biomass on environmental DNA shedding, degradation, and size distribution. Ecol. Evol..

[25] Piggott MP (2016). Evaluating the effects of laboratory protocols on eDNA detection probability for an endangered freshwater fish. Ecol. Evol..

[26] Deiner K, Altermatt F (2014). Transport distance of invertebrate environmental DNA in a natural river. PLoS One.

[27] Jane SF (2014). Distance, flow and PCR inhibition: eDNA dynamics in two headwater streams. Mol. Ecol. Resour..

[28] Shogren AJ, Tank JL, Andruszkiewicz E, Olds B, Mahon AR, Jerde CL, Bolster D (2017). Controls on eDNA movement in streams: Transport, retention, and resuspension. Sci. Rep..

[29] Klymus KE, Richter CA, Chapman DC, Paukert C (2015). Quantification of eDNA shedding rates from invasive bighead carp *Hypophthalmichthys nobilis* and silver carp *Hypophthalmichthys molitrix*. Biol. Conserv..

[30] Stoeckle MY, Soboleva L, Charlop-Powers Z (2017). Aquatic environmental DNA detects seasonal fish abundance and habitat preference in an urban estuary. PLoS One.

[31] Lacoursière-Roussel A, Rosabal M, Bernatchez L (2016). Estimating fish abundance and biomass from eDNA concentrations: Variability among capture methods and environmental conditions. Mol. Ecol. Resour..

[32] Moyer GR, Díaz-Ferguson E, Hill JE, Shea C (2014). Assessing environmental DNA detection in controlled lentic systems. PLoS One.

[33] Takahara T, Minamoto T, Yamanaka H, Doi H, Kawabata Z (2012). Estimation of fish biomass using environmental DNA. PLoS One.

[34] Kreader CA (1998). Persistence of PCR-detectable *Bacteroides distasonis* from human feces in river water. Appl. Environ. Microbiol..

[35] Bochove (2020). Organic matter reduces the amount of detectable environmental DNA in freshwater. Ecol. Evol..

[36] Mächler E, Osathanunkul M, Altermatt F (2018). Shedding light on eDNA: Neither natural levels of UV radiation nor the presence of a filter feeder affect eDNA-based detection of aquatic organisms. PLoS One.

[37] Monroe EM, Lynch C, Soluk DA, Britten HB (2010). Nonlethal tissue sampling techniques and microsatellite markers used for first report of genetic diversity in two populations of the endangered *Somatochlora hineana* (Odonata: Corduliidae). Ann. Entomol. Soc. Am..

[38] Schmidt, K. J. *Developing eDNA Techniques for the Endangered Hine’s Emerald Dragonfly (Somatochlora hineana) and Its Symbiont the Devil Crayfish (Cambarus [=Lacunicambarus] Diogenes): Mesocosm and Field Studies*. (University of South Dakota, 2020).

[39] Jackson C, McCalla SG, Amberg J, Soluk DA, Britten HB (2018). The complete mitochondrial genome of Hine’s emerald dragonfly (*Somatochlora hineana* Williamson) via NGS sequencing. Mitochondrial DNA Part B.

[40] Wickham H (2009). ggplot2: Elegant Graphics for Data Analysis.

[41] Klymus KE (2020). Reporting limits of detection and quantification for environmental DNA assays. Environ. DNA..

[42] R Core Team. *R: A Language and Environment for Statistical Computing*. (R Foundation for Statistical Computing, 2020). http://www.R-project.org/.

[43] Bates D, Maechler M, Bolker B, Walker S (2015). Fitting linear mixed-effects models using lme4. J. Stat. Softw..

[44] Pinheiro, J. C. & Bates, D. M. Fitting nonlinear mixed-effects models. In *S and S-Plus* (eds. Pinheiro, J.C. & Bates, D. M.) 337–414. (Springer, 2000).

